# Fulminant multi-organ myositis as a rare immune-related adverse event of nivolumab–relatlimab (Opdualag) therapy in metastatic melanoma: a case report

**DOI:** 10.3389/fimmu.2026.1721711

**Published:** 2026-03-02

**Authors:** Adi Moyal, Elimelech Rosenberg, Mohamed Asale, Odelya Peretz, Nikita Povlaive, May Shimshon Turgeman, Alexander Yakobson

**Affiliations:** 1 Department of Internal Medicine, Soroka University Medical Center, Beer-Sheva, Israel; 2 The Legacy Heitage Cancer Center, Dr. Larry Norton Institute, Soroka University Medical Center, Beer-Sheva, Israel; 3 Faculty of Health Sciences, Ben-Gurion University of the Negev, Beer-Sheva, Israel

**Keywords:** immune checkpoint inhibitors (ICIs), immune-related adverse events (irAEs), metastatic melanoma, myositis, Opdualag (nivolumab–relatlimab)

## Abstract

Immune checkpoint inhibitors (ICIs) have transformed the management of metastatic melanoma, providing durable survival benefits in a subset of patients. However, their use is associated with immune-related adverse events (irAEs), which may be unpredictable, severe, and occasionally fatal. We describe the case of a 77-year-old man with metastatic melanoma treated with nivolumab plus relatlimab (Opdualag), who achieved a marked antitumor response after seven treatment cycles. He subsequently developed fulminant late-onset myositis with multi-organ involvement, representing a particularly severe and atypical presentation. Despite timely recognition and aggressive immunosuppressive therapy, the clinical course was unfavorable, and the patient ultimately succumbed to these complications. To the best of our knowledge, this is the first published report of such a rare and fulminant multi-organ presentation of Opdualag-associated myositis. This report highlights the importance of vigilance for delayed and life-threatening irAEs and underscores the need for interdisciplinary collaboration and structured monitoring protocols as novel ICI combinations enter routine clinical practice.

## Introduction

Immune checkpoint inhibitors (ICIs) are monoclonal antibodies that target inhibitory immune regulators such as CTLA-4 (e.g., ipilimumab), PD-1 (e.g., nivolumab and pembrolizumab), PD-L1 (e.g., atezolizumab and durvalumab), and LAG-3 (e.g., relatlimab and fianlimab), thereby enhancing T-cell activation and promoting antitumor immune responses (1). These agents have transformed the therapeutic landscape of several advanced malignancies by disrupting the immune tolerance mechanisms used by tumors to evade detection.

In metastatic melanoma, ICIs have become a cornerstone of first-line systemic therapy, significantly improving overall survival and durable disease control in a subset of patients (2). Despite their efficacy, ICIs are associated with immune-related adverse events (irAEs), including inflammatory toxicities that result from non-specific immune activation that can affect multiple organ systems, including the skin, gastrointestinal tract, liver, endocrine glands, and muscles (3).

Opdualag, a fixed-dose combination of nivolumab (anti-PD-1) and relatlimab (anti-LAG-3), was recently approved as a first-line treatment for metastatic melanoma after demonstrating superior efficacy compared to PD-1 inhibition monotherapy (4). As with other ICIs, its use may result in irAEs, potentially affecting various organ systems and ranging in severity.

## Case presentation

### Background, staging, and immunotherapy response

A 77-year-old man with a medical history of ischemic heart disease (underwent coronary artery bypass grafting in 2008), hypertension, and well-controlled type 2 diabetes has been under oncologic follow-up since 2018 for localized melanoma of the right forearm, which was completely excised. The tumor was staged as T3bN0M0, indicating a high risk of recurrence despite the absence of nodal or distant metastasis at diagnosis.

In March 2024, a biopsy of a new soft tissue lesion in the left lower back was performed, revealing metastatic malignant melanoma. A staging CT total body (CTTB) demonstrated extensive metastatic spread to the lungs, mediastinum, liver, peritoneum, pancreas, and soft tissue. A brain MRI revealed a left temporal hemorrhage and supratentorial parenchymal metastases. Molecular testing confirmed a BRAF wild-type status.

**Figure 1:**
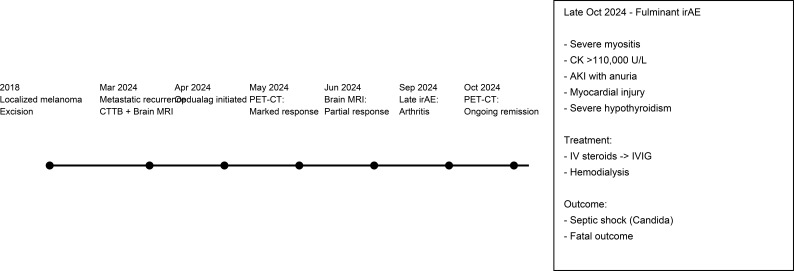
Clinical timeline illustrating the oncologic course and treatment response prior to the fulminant irAEs, followed by a schematic summary of the multi-organ immune-related toxicity, management, and outcome. irAEs, immune-related adverse events.

In April 2024, he initiated immunotherapy with Opdualag (nivolumab + relatlimab) once every 4 weeks. After only two cycles of immunotherapy, a PET–CT in May 2024 demonstrated a marked metabolic response with significant regression of metastatic lesions, including complete resolution of an abdominal wall metastasis. He completed a total of seven cycles of Opdualag, with overall good tolerance (**Figure 1**). Throughout the treatment period, he remained under close clinical and laboratory monitoring. Viral hepatitis serologic tests were negative prior to the initiation of immunotherapy, and thyroid function tests remained within normal limits during the entire treatment course. It was only in September 2024, near the completion of therapy, that he reported persistent muscle pain and was diagnosed with immune-related arthritis, consistent with a late-onset irAEs. Despite this, treatment response remained favorable, where a brain MRI in June 2024 showed a good partial response with no new metastases, and a follow-up PET–CT in October 2024 confirmed continued metabolic remission.

### Acute presentation and initial evaluation

In late October 2024, the patient presented to the emergency department with several days of anuria, vomiting, constipation, and progressive generalized muscle weakness. On examination, he appeared pale and fatigued but was afebrile and hemodynamically stable. Laboratory tests revealed acute kidney injury (creatinine 10 mg/dL and urea 180 mg/dL), mild leukocytosis, hepatocellular liver enzyme elevation, and markedly elevated Lactate Dehydrogenase (LDH) (600 U/L).

Further laboratory evaluation showed a Creatine Kinase (CK) of 110,323 U/L, myoglobin levels of 112,365 ng/mL, and troponin-T at 1,547 ng/L. An infectious disease workup (Hepatitis B Virus (HBV) and Hepatitis C Virus (HCV), HIV, and blood cultures) and autoimmune serology (Antinuclear Antibody (ANA), Antineutrophil Cytoplasmic Antibody (ANCA), and Rheumatoid Factor (RF)) were negative. Thyroid tests revealed hypothyroidism (Thyroid Stimulating Hormone (TSH) 36 mIU/L, T3 0.39 pg/mL, T4 0.25 ng/dL) and hyperparathyroidism (Parathyroid Hormone (PTH) 703 pg/mL). A thyroid gland ultrasound Doppler revealed an atrophic thyroid without blood flow. Urinalysis demonstrated nephrotic-range proteinuria (40 g/day), and urine microscopic examination showed Red Blood Cells (RBCs) without casts. CT scan of the abdomen revealed normal-sized kidneys without hydronephrosis. Echocardiography demonstrated preserved cardiac function.

### Diagnostic assessment and differential diagnosis

Given the fulminant presentation with extreme CK level and multi-organ involvement, a broad spectrum of differential diagnoses were systematically assessed. Statin-induced myopathy is a well-recognized side effect, but it rarely causes severe rhabdomyolysis years after the initiation of treatment. Myxedema can cause myopathy with significant CK elevation, but massive rhabdomyolysis is an exceptional phenomenon, with approximately 30 to 40 cases reported in the medical literature (5, 6). Nevertheless, intravenous levothyroxine was administered daily. Infectious myositis was rejected on a clinical basis. Overlap syndromes, including immune checkpoint inhibitor-associated myositis–myocarditis–myasthenia overlap syndrome, were clinically assessed; however, the patient did not exhibit ptosis, bulbar symptoms, fluctuating weakness, or other features suggestive of myasthenia gravis. The clinical presentation was consistent with ICI-induced severe myositis, complicated by rhabdomyolysis, acute renal failure, and likely myocardial involvement. Due to rapid clinical deterioration and progression to critical illness, further neuromuscular tests and diagnostic procedures, including electromyography, muscle biopsy, and cardiac MRI, were not feasible.

### Treatment course, ICU complications, and outcome

High-dose intravenous corticosteroids (pulse methylprednisolone 1,000 mg/day) were initiated 2 days post-hospital admission, along with hemodialysis for volume and electrolyte management. This approach was consistent with current expert recommendations advocating early aggressive immunosuppression for severe immune-related neuromuscular toxicities. Gradual clinical and laboratory improvement followed: the patient resumed minimal oral intake and bowel movements, while CK declined to 92,849 U/L and creatinine to 7.4 mg/dL, although anuria persisted.

Due to insufficient response, intravenous immunoglobulin (IVIG; 2 g/kg total administered over 2–5 days) was added to the high-dose steroid regimen following multidisciplinary consultation; plasma exchange was considered as an alternative escalation strategy, and IVIG was selected based on expert input from immunology and rheumatology teams. After 3 days of IVIG, laboratory results improved: CK decreased to 32,556 U/L, LDH to 1,859 U/L, and creatinine to 6.4 mg/dL. However, 36 hours post-IVIG completion, the patient developed septic shock. He was sedated and intubated, and broad-spectrum antibiotics were initiated. Blood cultures returned positive for *Candida* species. During his ICU stay, he developed atrial fibrillation requiring cardioversion. Despite maximal supportive therapy, he died 1 week later under mechanical ventilation and vasopressor support.

## Discussion

ICIs have revolutionized the cancer treatment landscape, particularly in melanoma, by enhancing T cell-mediated antitumor immunity through blockade of inhibitory receptors such as CTLA-4, PD-1, PD-L1, and LAG-3. The use of these agents, however, is associated with a broad spectrum of irAEs, which result from non-specific immune activation and can affect nearly any organ system (7). While dermatologic, endocrine, and gastrointestinal irAEs are most common, musculoskeletal manifestations such as myositis are rare, occurring in less than 1% of patients (7, 8).

ICI-induced myositis is rare and potentially life-threatening. It differs from classic idiopathic inflammatory myopathies in its T cell-predominant pathology, with minimal autoantibody involvement and frequent histologic evidence of CD8+ lymphocytic infiltration (8). A particularly severe phenotype, known as IM3OS (immune checkpoint inhibitor-associated myositis, myocarditis, and myasthenia gravis overlap syndrome), has been described and characterized by rapidly progressive weakness, bulbar and respiratory involvement, arrhythmias, and high mortality rates—reported in some cases to exceed 50% (9).

The case described herein presents a particularly severe and atypical manifestation of ICI-induced myositis related to Opdualag therapy. Opdualag is a relatively new combination drug consisting of anti-PD-1 and anti-LAG-3 antibodies that was approved for metastatic melanoma in 2022. There are several notable features in this case report that deviate from the typical presentation of ICI-induced myositis described in the literature. First, to date, serious irAEs using this regimen have been limited mainly to common toxicities such as musculoskeletal pain and endocrine disturbances, with no robust reports of fulminant myositis in the pivotal RELATIVITY-047 trial (4). Second, the onset of symptoms in our patient occurred after seven cycles, significantly later than the 4–8-week window commonly observed with other ICIs (8) (9). This especially highlights the need for vigilance throughout treatment, even in patients showing clinical benefit.

Additionally, the patient has significantly elevated CK levels (>110,000 U/L), which are among the highest reported, where most ICI-induced myopathy cases report CK elevations <10,000 U/L, with levels rarely exceeding 50,000 U/L (8). The disease course was further complicated by multi-organ involvement, including rhabdomyolysis-induced acute kidney injury, requiring dialysis, severe hypothyroidism, and myocardial injury—an aggressive systemic picture contrasting with reports focusing on isolated muscle or mild cardiac involvement (8, 9). Although high-dose corticosteroids and IVIG were promptly administered, clinical and laboratory responses were only partial, and the patient ultimately deteriorated. This underscores another clinical challenge of managing fulminant irAEs: aggressive immunosuppression is necessary but must be balanced against infection risk, a dilemma emphasized by recent American Society of Clinical Oncology (ASCO) / European Society for Medical Oncology (ESMO) guidelines (10).

This case serves as a reminder of the diagnostic and therapeutic complexity posed by immune checkpoint inhibitor-related toxicities. It illustrates how severe irAEs may develop late in the treatment course, mimic or overlap with multiple disease processes, and rapidly progress despite appropriate immunosuppressive therapy. For clinicians, particularly those outside oncology, awareness of such rare but critical complications is essential. Early recognition, timely escalation of care, and multidisciplinary coordination remain the cornerstones of successful management. This case also highlights existing gaps in clinical guidance for managing atypical or fulminant irAEs, emphasizing the need for continued research and refined clinical protocols tailored to real-world complexities.

## Conclusion

ICI-induced myositis remains a rare but serious clinical entity, especially when presenting with delayed onset and multi-organ involvement. As the use of immunotherapy becomes increasingly prominent, the recognition and management of irAEs require heightened awareness beyond the oncology community. This case underscores the importance of interdisciplinary collaboration, particularly between oncologists and internists, in identifying and responding to atypical and severe toxicities such as ICI-induced myositis. Structured monitoring protocols and education on delayed irAEs are essential to ensure timely diagnosis and optimal patient outcomes.

To the best of our knowledge, this is the first published report of such a rare and fulminant case of multi-organ myositis associated with Opdualag. As newer ICI combinations become integrated into routine clinical practice, such reports are essential to expand the collective understanding of rare immune-related toxicities and to guide timely recognition and intervention.

## Data Availability

The original contributions presented in the study are included in the article/supplementary material. Further inquiries can be directed to the corresponding author.
